# Association between depression and the risk of Alzheimer’s disease using the Korean National Health Insurance Service-Elderly Cohort

**DOI:** 10.1038/s41598-021-02201-6

**Published:** 2021-11-19

**Authors:** Hyunkyu Kim, Wonjeong Jeong, Junhyun Kwon, Youseok Kim, Eun-Cheol Park, Sung-In Jang

**Affiliations:** 1 Department of Preventive Medicine and Institute of Health Services Research, Yonsei University College of Medicine, 50 Yonsei-ro, Seodaemun-gu, Seoul, 03722 Republic of Korea; 2 Institute of Health Services Research, Yonsei University, Seoul, Republic of Korea; 3 Department of Psychiatry, Yonsei University College of Medicine, Seoul, Republic of Korea; 4 Department of Public Health, Graduate School, Yonsei University, Seoul, Republic of Korea; 5 Department of Hospital Administration, Graduate School of Public Health, Yonsei University, Seoul, Republic of Korea

**Keywords:** Depression, Dementia

## Abstract

In this cohort study, we assessed the association between depression and the risk of Alzheimer’s disease from data obtained from the 2002 to 2013 Korean National Health Insurance Service-Elderly Cohort Database, which accounts for 10% of the South Korean population aged > 60 years. A total 518,466 patients were included in the analysis and followed up, unless they were excluded due to death or migration. Patients who sought treatment for depression or dementia within 1 year of the washout period and who were diagnosed with dementia within the 1-year period of the diagnosis of depression were excluded from the study. The risk of dementia was analysed using Cox proportional hazards models. Patients with a history of depression during the follow-up period were at a higher risk of Alzheimer’s disease than those without a history of depression (HR 3.35, CI 3.27–3.42). The severe-depression group exhibited the highest risk of Alzheimer’s disease (HR 4.41, CI 4.04–4.81), while the mild-depression group exhibited a relatively lower risk of Alzheimer’s disease (HR 3.31, CI 3.16–3.47). The risk of Alzheimer’s disease was associated with depression history and an increased severity of depression increased the risk of Alzheimer’s disease.

## Introduction

Dementia has become a major health issue as a result of the progression of the ageing process, with repercussions such as cognitive impairment and deterioration in the ability to perform daily activities^1,2^. Cognitive impairment caused by dementia places a major socio-economic burden over patients and their families owing to the poor quality of life, hospitalisation, increased mortality, and poverty^3–8^. Several studies have focused on early detection and intervention for cognitive decline, to alleviate the financial and public health impact of the surge in the incidence of dementia^9^. Other studies have focused on ascertaining the conditions associated with the incidence of dementia, that is, preventing dementia by impeding the occurrence of associated conditions, such as hypertension and diabetes^10–12^. The subtypes of dementia should also be considered for prevention due to their difference in etiology^13,14^. Among the types of dementia, Alzheimer’s Disease was the most prevalent type (2.1–6.4%) and followed by Vascular Dementia (0.1–2.9%), Mixed Dementia (0.2–1.4%) in four studies^15^.

Neuropsychiatric conditions, including depression, insomnia, and drug use, are among the conditions associated with dementia, and were found to be associated with cognitive impairment^16–18^. Previous studies demonstrated that depression frequently occurs in concomitance with dementia, suggesting that the possibility of an association between these conditions and a shared common aetiology^19^. One meta-analysis suggested that dementia was attributed to depression in 7.9% of the worldwide depression population and 11.1% of that in the USA^20^. These results suggest that managing depression may limit the occurrence of dementia, which can curtail the socio-economic cost of treating patients with dementia. Therefore, investigating the causal relationship between depression and dementia is critical. There are several previous cohort studies suggesting that depression might be a risk factor for dementia^21^. The confirmation of the association between depression and the risk of dementia in nationally representative cohort data would provide a strong cornerstone for the prophylaxis of dementia through the modulation and management of depression in the elderly population. Moreover, if the risk of dementia increases with the increase in severity of depression, it would support the causality between the two conditions. We used Alzheimer’s disease to represent dementia because it contributes to over 70% of dementia cases in Korea. Moreover, we wished to exclude any external causes of dementia, including trauma.

The aim of this study was to investigate the association between depression and the risk of Alzheimer’s disease in an elderly Korean cohort, using the national population based cohort from South Korean National Health Insurance claims data after adjusting for covariates assumed to affect cognitive function. Moreover, we further investigated the difference in the risk of Alzheimer’s disease depending on the severity of depression to elucidate the intensity-dependent association between depression and dementia.

## Materials and methods

### Study population and data

The data analysed in this study were acquired from the Korean National Health Insurance Service-Elderly Cohort Database (NHIS-ECD) of the National Health Insurance Service (NHIS) between 2002 and 2013. The Korean NHIS provides researchers with all data on the claims collected under the NHIS for the purpose of academic investigation and policy making. The NHIS-ECD, one of the sample cohorts provided by the NHIS, includes all medical claims from 558,147 elderly health insurance beneficiaries and medical benefit entitlement holders, which accounts for 10% of South Korean population aged over 60 years by random sampling. The patients in the cohort were followed up, unless he/she was excluded due to death or migration. The NHIS-ECD database includes information on the socio-economic status and the clinically determined International Classification of Disease, 10th revision (ICD-10) codes. The NHIS-ECD data were de-identified and thus the need for informed consent was waived by the Institutional Review Board.

**Figure 1:**
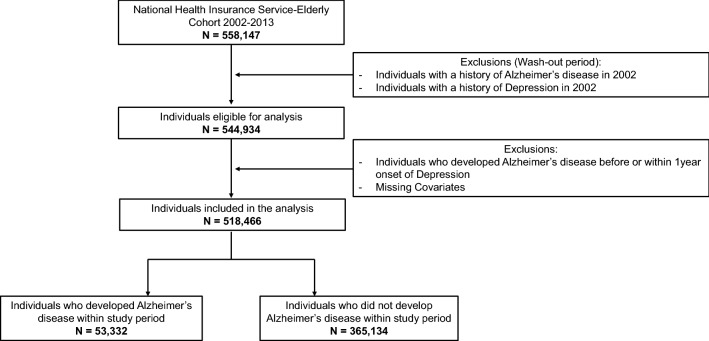
Flowchart of the participant selection.

Patients who sought treatment for depression or dementia in the wash-out period of the cohort data was excluded. The first year of cohort data was designated as the wash-out period since the treatment in the first year might be the consequence of previously existing diseases and this study aimed the newly onset depression and Alzheimer’s disease. Moreover, patients who were diagnosed with Alzheimer’s disease within the 1-year period of the diagnosis of depression or diagnosed Alzheimer’s disease before the diagnosis of depression were excluded from the study (Fig. 1).

### Study variables and covariates

Patients were diagnosed to have Alzheimer’s disease based on the diagnostic ICD-10 codes F00 and G30 at the first visit. We defined patients with depression by using the ICD-10 codes F32 and F33. The first visit with the diagnostic code F32, F33 were regarded as the diagnostic date of Depression. The severity of the depression was defined by the ICD-code. We divided the patients with depression into subgroups using the ICD-10 code: F32.0 was designated as mild depression, F32.1 as moderate depression, and F32.2 and F32.3 as severe depression, to investigate the difference in the risk of Alzheimer’s disease among the various groups with different severities of depression.

Baseline demographic information including age, sex, health insurance status, residential region, disability, income status and comorbid status including the Charlson comorbidity index (CCI) were included in the regression model as covariates. Health insurance system was categorized into two groups: health insurance, medical aid^22^. Based on the criteria of Korea’s health insurance system, people should register the National Health Insurance Service unless they receive the Medical Aid beneficiaries which is for the people whose income level is below the government-defined poverty level. CCI was used to identify the patients’ comorbidities^23^.

### Statistical analysis

Chi-squared tests were used to investigate and compare the general characteristics of the study population. A Cox proportional hazards model was generated to examine the association between depression and the risk of dementia in the participants. The covariates were included in the analysis. Independent subgroup analyses were performed to investigate the combined effects of depression and other covariates on Alzheimer’s disease. We divided patients with depression into three groups and analysed them using the Cox proportional hazards model to investigate the dependency of the risk of dementia on the severity of depression. The results were presented as hazard ratios (HRs) and confidence intervals (CIs) to compare the risk of Alzheimer’s disease among the groups. All analyses were conducted using SAS software version 9.4 (SAS Institute, Cary, North Carolina, USA). *P*-values < 0.05 were considered statistically significant.

### Ethical considerations

The database we used in this study was based on routinely collected administrative and claims data. As the NHIS-ECD data do not contain any identifying information, the study was approved as exempt by the Institutional Review Board of Yonsei University’s Health System. This study adhered to the tenets of the Declaration of Helsinki.

## Results

The general characteristics of the study population are presented in Table 1. A total of 518,466 patients were included in the analysis: 10.3% of patients were diagnosed with dementia during the follow-up period, while 14.9% of patients were diagnosed with depression. The chi-squared test revealed that the incidence of Alzheimer’s disease was significantly higher in patients with depression than in those without depression. Sex, age, social security status, residential region, disability status, income, and CCI were also identified as having statistically significant effects on the risk of Alzheimer’s disease.

**Table 1:** General characteristics of study population and chi-squared test results for analysing Alzheimer’s disease risk.

Variables	Total	Risk of Alzheimer’s disease		*P*-value
		Yes	No	
Total	518,466 (100.0)	53,332 (10.3)	465,134 (89.7)	
**Depression**				< 0.0001
Present	77,013 (14.9)	11,335 (14.7)	65,678 (85.3)	
Absent	441,453 (85.1)	41,997 (9.5)	399,456 (90.5)	
**Sex**				< 0.0001
Male	217,649 (42.0)	15,884 (7.3)	201,765 (92.7)	
Female	300,817 (58.0)	37,448 (12.4)	263,369 (87.6)	
**Age (years)**				< 0.0001
< 65	9880 (1.9)	242 (2.4)	9638 (97.6)	
65–70	203,196 (39.2)	10,579 (5.2)	192,617 (94.8)	
70–75	139,572 (26.9)	13,348 (9.6)	126,224 (90.4)	
75–80	90,684 (17.5)	13,620 (15.0)	77,064 (85.0)	
≥ 80	75,134 (14.5)	15,543 (20.7)	59,591 (79.3)	
**Social security**				< 0.0001
Health insurance	467,996 (90.3)	44,406 (9.5)	423,590 (90.5)	
Medical aid	50,470 (9.7)	8926 (17.7)	41,544 (82.3)	
**Region**				< 0.0001
Metropolitan	180,480 (34.8)	15,876 (8.8)	164,604 (91.2)	
City	114,040 (22.0)	12,530 (11.0)	101,510 (89.0)	
Rural	223,946 (43.2)	24,926 (11.1)	199,020 (88.9)	
**Disability**				< 0.0001
Yes	6896 (1.3)	1269 (18.4)	5627 (81.6)	
No	511,570 (98.7)	52,063 (10.2)	459,507 (89.8)	
**Income**				< 0.0001
Low	126,134 (24.3)	16,498 (13.1)	109,636 (86.9)	
Middle	155,700 (30.0)	13,795 (8.9)	141,905 (91.1)	
High	236,632 (45.6)	23,039 (9.7)	213,593 (90.3)	
**Charlson comorbidity index (CCI)**				< 0.0001
0–2	148,099 (28.6)	7164 (4.8)	140,935 (95.2)	
3–4	166,189 (32.1)	13,742 (8.3)	152,447 (91.7)	
≥ 5	204,178 (39.4)	32,426 (15.9)	171,752 (84.1)	

Variables are presented as numbers and percentages.

Table 2 shows the results of the Cox proportional hazards regression analysis for the association between depression and the risk of Alzheimer’s disease after adjusting for the above-mentioned covariates. Individuals with a history of depression during the follow-up period were at a higher risk of developing Alzheimer’s disease than those without a history of depression (HR 3.35, CI 3.27–3.42). Women were at a higher risk of Alzheimer’s disease compare to men (HR 1.32, CI 1.29–1.34). The social security status was also a statistically significant factor affecting the risk of Alzheimer’s disease: patients with medical aid possessed a higher risk than patients with health insurance (HR 2.09, CI 2.03–2.16). Other covariates that were statistically significantly associated with the risk of dementia as shown in Table 2.

**Table 2:** Cox proportional hazards regression analysis results for the association between depression and Alzheimer’s disease risk.

Variables	Alzheimer’s disease	
	HR	95% CI
**Depression**		
Present	3.35	(3.27–3.42)
Absent	1.00	
**Sex**		
Male	1.00	
Female	1.32	(1.29–1.34)
**Age (years)**		
< 65	1.00	
65–70	1.37	(1.21–1.56)
70–75	2.35	(2.07–2.67)
75–80	4.04	(3.56–4.59)
≥ 80	7.07	(6.22–8.02)
**Social security**		
Health insurance	1.00	
Medical aid	2.09	(2.03–2.16)
**Region**		
Metropolitan	1.00	
City	1.27	(1.24–1.30)
Rural	1.22	(1.19–1.24)
**Disability**		
Yes	1.35	(1.28–1.43)
No	1.00	
**Income**		
Low	1.00	
Middle	0.95	(0.92–0.97)
High	0.96	(0.94–0.99)
**Charlson comorbidity index (CCI)**		
0–2	1.00	
3–4	1.46	(1.42–1.51)
≥ 5	2.91	(2.83–2.99)

Independent subgroup analyses were conducted to assess the combined effects of depression and other sociodemographic variables on the risk of Alzheimer’s disease, as shown in Table 3. Patients with depression showed a higher risk amongst all the subgroups, which were divided by sex, age, social security status, region, income, disability, and CCI. In the subgroup analysis by age, the youngest group exhibited the highest HR compared to the older age groups.

**Table 3:** Subgroup analysis of association between Alzheimer’s disease risk and covariates, according to history of depression.

	No depression	Depression	
	Adjusted HR	Adjusted HR	95% CI
**Sex**			
Male	1.00	3.75	(3.59–3.91)
Female	1.00	3.22	(3.14–3.30)
**Age (years)**			
< 65	1.00	4.93	(3.67–6.22)
65–70	1.00	3.94	(3.76–4.12)
70–75	1.00	3.58	(3.43–3.73)
75–80	1.00	3.09	(2.96–3.23)
≥ 80	1.00	2.82	(2.69–2.96)
**Social security**			
Health insurance	1.00	3.51	(3.43–3.59)
Medical aid	1.00	2.63	(2.49–2.77)
**Region**			
Metropolitan	1.00	3.51	(3.38–3.65)
City	1.00	3.41	(3.26–3.57)
Rural	1.00	3.19	(3.09–3.30)
**Income**			
Low	1.00	2.96	(2.84–3.08)
Middle	1.00	3.59	(3.44–3.75)
High	1.00	3.50	(3.39–3.61)
**Disability**			
Yes	1.00	2.75	(2.38–3.19)
No	1.00	3.36	(3.29–3.43)
**Charlson comorbidity index (CCI)**			
0–2	1.00	3.18	(2.93–3.45)
3–4	1.00	2.99	(2.85–3.14)
≥ 5	1.00	3.56	(3.47–3.66)

Table 4 shows the results of the subgroup analysis based on the severity of depression and the risk of Alzheimer’s disease throughout the study period. The mild-depression group showed a relatively lower risk of Alzheimer’s disease (HR 3.31, CI 3.16–3.47) and the severe-depression group showed the highest risk of Alzheimer’s disease (HR 4.41, CI 4.04–4.81).

**Table 4:** Results of the association between depression and the risk of Alzheimer’s disease.

Variables	Alzheimer’s disease	
	HR	95% CI
**Depression**		
No	1.00	
Mild	3.31	(3.16–3.47)
Moderate	3.75	(3.55–3.97)
Severe	4.41	(4.04–4.81)

The results were obtained after adjusting all the covariates included in the regression model in Table 2.

## Discussion

The current study found that the history of depression was associated with the risk of Alzheimer’s disease in an elderly Korean cohort over a 12-year follow-up period. Furthermore, we found a severity-dependent elevation in the risk of Alzheimer’s disease based on the severity of depression diagnosed at the first visit.

Previous studies have demonstrated the association between depression and the risk of Alzheimer’s disease, which are generally in agreement with our results. Holmquist et al. reported that the diagnosis of depression was associated with dementia (OR 2.47) in the Swedish National Cohort^24^. Kanton et al. reported that patients with type 2 diabetes with depression possessed a higher risk of dementia (HR 2.02) than those with only diabetes (i.e., the control group)^25^. The risk of the dementia was relatively lower compared to our main results (HR 3.35), but the higher HR of our results may be reasonable considering the older age of the participants and longer follow-up period of our study.

In the subgroup analysis, people with medical aid showed lower risk elevation (HR 2.63) than people with Health insurance (HR 3.51). The result is similar after subgrouping subjects by income tertile that low income group showed lowest risk elevation among the three income level groups (HR 2.96). These results probably due to the distinct association between socioeconomic status and dementia that low income group already had higher prevalence of Alzheimer’s disease, thus depression had lower risk elevation than higher income groups^26^. Additionally, Petersen et al. suggested that higher income people seems to receive dementia diagnosis earlier than lower socioeconomic status group which can also explain our results^27^.

The causality between depression and the risk of Alzheimer’s disease is currently being investigated by several study groups. Holmquist et al. compared the short-term association and long-term association of depression with dementia, and found that the history of depression retains its association with dementia 20 years after the diagnosis^24^. This finding may provide evidence that the history of depression remains a risk factor of dementia throughout the patient's lifetime. Another approach to elucidate the causal association between depression and dementia entails measuring the intensity-effect modulating the relationship. One study performed with extremely old women found that the cumulative effect of depressive symptoms was associated with the development of dementia or mild cognitive impairment^28^. Other studies attempted to reveal that severe depression greatly increased the risk of dementia compared to milder depression with tentative results; the explanation of causality was limited owing to the short follow-up period^29,30^. The increase in the HR in this study with the increase in the severity of depression at the first diagnosis reflected the elevation of the risk of Alzheimer’s disease, which is consistent with previous studies. Our study provided more evidence of the severity-dependent increase in the risk of dementia among patients with depression.

The mechanism by which depression increases the risk of Alzheimer’s disease is still unclear, although previous studies have suggested some possible hypotheses. Hippocampal atrophy is one of the possible reasons why a history of depression influences the development of Alzheimer’s disease. Magnetic resonance imaging studies have found a reduction in the volume of the hippocampus in patients with depression; moreover, late-onset depression showed a stronger association with hippocampal atrophy^31,32^. Another possible explanation is that both depression and Alzheimer’s disease share a common aetiology. Oxidative stress results in brain damage that triggers the antioxidant defences, including amyloid-β elevation and increased hyper-phosphorylation of tau, both of which occur in major depressive disorder and Alzheimer’s disease^33^. These results suggest a possible explanation that the molecular pathways may occur in earlier life as a depressive symptom and occur as dementia symptoms. Social isolation, due to the depression, may affect the occurrence of dementia. The severe depressive symptoms including depressed mood, fatigue, helplessness restrict patients from interacting with other people result in social isolation and feeling loneliness. Both social isolation and loneliness increases the risk of dementia thus depression could increase the risk of dementia^34,35^.

This study has several limitations. First, known conditions that could affect the development of Alzheimer’s disease, such as a family history of depression or dementia^36^ and the individual’s alcohol consumption could not be assessed, owing to the retrospective design of the study^37^. Second, the number of patients diagnosed with depression might be underestimated in the analysis since the study used claims data. Patients with depressive symptoms are unwilling to visit clinics or hospitals as psychiatric disorders and treatments are often regarded as a cultural taboo in Korea; thus, the number of patients analysed could be lower than the actual population with depression. Third, the accuracy of the diagnostic information may be limited, owing to the inaccuracy of the claims diagnosis, as suggested by a previous study^38^. We investigated the primary and secondary diagnostic codes and included both in the analysis to increase the accuracy of the diagnosis.

Despite these limitations, our study possesses certain strengths. Although the use of claims data is associated with limitations, we used national sampling cohort data that represent 10% of the elderly population in Korea. The results of our study can be generalised to the entire population of elderly South Korean individuals and may provide a background for the management of depression in adulthood to curb the future incidence of Alzheimer’s disease. Moreover, since we only included patients with new-onset depression, this study provides additional evidence that new-onset depression in the elderly is a risk factor for Alzheimer’s disease, and the severity of depression at the first visit is an important predictor of the development of dementia in these patients.

In conclusion, this study identified the association between the history of depression and the risk of Alzheimer’s disease in the elderly Korean population and observed a severity-dependent association with the risk of Alzheimer’s disease. Further research using prospective designs that allow for a clear elucidation of causality of depression-induced dementia in a controlled environment should be conducted to validate these findings.
